# Reliability and Stability Issues in Bi_2_O_2_Se/β-Bi_2_SeO_5_ Field-Effect Transistors

**DOI:** 10.1021/acsnano.6c00419

**Published:** 2026-07-14

**Authors:** Mina Bahrami, Mohammad Rasool Davoudi, Axel Verdianu, Pedram Khakbaz, Dominic Waldhoer, Christoph Wilhelmer, Junchuan Tang, Congwei Tan, Aftab Nazir, Yu Zheng, Changze Liu, Hailin Peng, Theresia Knobloch, Michael Waltl, Tibor Grasser

**Affiliations:** † Institute for Microelectronics, Technische Universität Wien, Vienna 1040, Austria; ‡ College of Chemistry and Molecular Engineering, 12465 Peking University, Beijing 100871, China; § Huawei Technologies Research and Development Belgium N.V., Leuven 3001, Belgium

**Keywords:** Bi_2_O_2_Se, β-Bi_2_SeO_5_, high-κ native oxide, 2D semiconductor, zipper materials, charge trapping, bias temperature instability (BTI)

## Abstract

The layered semiconductor Bi_2_O_2_Se is a promising candidate for next-generation nanoelectronic devices because it combines favorable electronic and structural properties. These include an exceptionally high electron mobility, a suitable bandgap and air stability, and most importantly, the ability to form a high-κ native oxide, Bi_2_SeO_5_ (with a calculated bulk κ > 30). However, intrinsic point defects in the semiconductor and the oxide can influence the electronic behavior and long-term reliability. In this work, we employ density functional theory to investigate the electronic structure, formation energies, trap levels, and relaxation energies of the relevant native defects in both Bi_2_O_2_Se and β-Bi_2_SeO_5_. Our results demonstrate that selenium and oxygen vacancies in the semiconductor Bi_2_O_2_Se act as shallow donors, donating free electrons to the conduction band, consistent with the commonly observed unintentional n-type conductivity. In contrast, oxygen vacancies in the dielectric β-Bi_2_SeO_5_ introduce deep-level trap states, indicating that they may act as performance-limiting charge trapping centers during device operation. We successfully correlate these theoretically identified defects with experimental device measurements from a prototype Bi_2_O_2_Se/β-Bi_2_SeO_5_ field-effect transistor, structurally characterized via scanning transmission electron microscopy. We finally demonstrate the contribution of defects to critical reliability issues, specifically hysteresis and bias temperature instability. These insights provide input for reliability modeling and defect engineering strategies for improving the long-term stability of Bi_2_O_2_Se/Bi_2_SeO_5_-based nanoelectronics.

## Graphical abstract

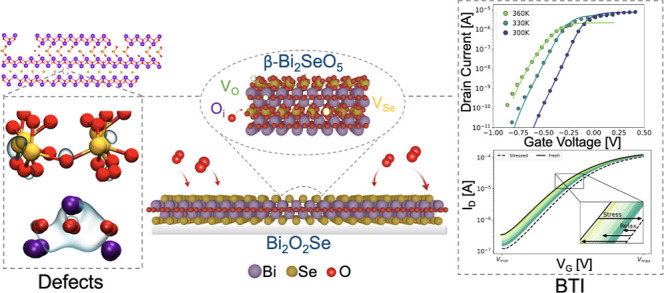

Layered materials such as graphene,^1–3^ transition-metal dichalcogenides,^4,5^ black phosphorus,^6^ and indium selenide^7,8^ exhibit a diverse range of unique physical properties. These properties enable a wide array of applications, including memory devices,^9–11^ optoelectronic devices,^12–14^ and field-effect transistors (FETs).^15,16^ Among these materials, the semiconducting layered compound Bi_2_O_2_Se has attracted growing attention due to its promising properties for electronic device applications, including excellent air stability. Even more importantly, Bi_2_O_2_Se exhibits an exceptionally high-measured Hall electron mobility (812 cm^2^ V^–1^ s^–1^ at room temperature^17^), considerably exceeding many other 2D semiconductors and relevant roadmap targets, a key property for high-performance electronic applications.^18^ High-quality ultrathin films of Bi_2_O_2_Se can be grown at relatively low temperatures via chemical vapor deposition (CVD), using Bi_2_O_3_ and Bi_2_Se_3_ as precursors and materials such as fluorophlogopite mica or strontium titanate as substrates.^17,19^ Moreover, the layered semiconductor can be prepared using other techniques, such as metal–organic chemical vapor deposition (MOCVD) and molecular beam epitaxy.^20^ Bi_2_O_2_Se possesses a body-centered tetragonal lattice with the *I*4/*mmm* space group, consisting of positively charged ${\left[{Bi}_{2}O_{2}\right]}_{n}^{2n+}$ layers and negatively charged [Se]_*n*_^2*n*–^ layers that are bound by electrostatic interactions.^21^ This layered structure has been referred to as a “zipper” 2D material due to the absence of a van der Waals (vdW) gap in its crystal structure and larger binding energies between layers compared to most vdW materials.^22^ The distinctive zipper-like structure of exfoliated surfaces of Bi_2_O_2_Se is characterized by 50% selenium (Se) coverage in each layer. This partial coverage results in the formation of surface Se vacancies, which tend to dimerize into a repeating 2 × *n* pattern.^20^ Notably, this arrangement creates a surface free of dangling bonds, contributing to its stability. Crucially, distinct from the surface vacancies that facilitate the zipper-like structure, selenium vacancies (V_Se_) are recognized as prominent native point defects present throughout the multilayer structure in the layered semiconductor Bi_2_O_2_Se. Within the material’s structure, selenium atoms occupy a unique plane and are linked by weak electrostatic interactions to the Bi_2_O_2_ layer, making vacancy formation energetically favorable.

A key advantage of Bi_2_O_2_Se over many other 2D semiconductors is its ability to form a compatible native oxide, bismuth oxoselenate (Bi_2_SeO_5_).^22–25^ Unlike many other 2D semiconductors that lack stable and high-quality native oxides, Bi_2_O_2_Se can be directly oxidized to form different phases of Bi_2_SeO_5_. Two distinct native oxide phases have been demonstrated so far: α-Bi_2_SeO_5_, typically synthesized through thermal oxidation,^24^ and β-Bi_2_SeO_5_, obtained via ultraviolet (UV)-assisted intercalative oxidation. During the UV-assisted process, the [Bi_2_O_2_] framework remains structurally intact, while the interlayer Se anions are oxidized, forming [SeO_3_] tetrahedra sandwiched between the [Bi_2_O_2_] layers,^23^ as illustrated in Figure 1a. This process results in an interlayer expansion along the *c*-axis from 6.08 Å in Bi_2_O_2_Se to about 7.51 Å in β-Bi_2_SeO_5_ ^22^ and preserves the single-crystalline nature and alignment with the underlying semiconductor. β-Bi_2_SeO_5_ stands out as a promising gate dielectric due to its ability to form an atomically flat and nearly lattice-matched interface with Bi_2_O_2_Se. This high-quality interface minimizes interface defects and scattering, which is essential for maintaining the large carrier mobility of the Bi_2_O_2_Se channel and achieving high-performance devices.^20,22^ Additionally, β-Bi_2_SeO_5_ exhibits a high bulk dielectric constant, with a calculated value exceeding 30 and an experimentally reported value of 22 for 3–6 layers,^22,23^ along with low leakage currents and favorable band alignment with Bi_2_O_2_Se (calculated conduction-band offset: 1.55–1.86 eV; valence-band offset: 1.13–1.21 eV^22^), making it a promising candidate for future transistor technologies.

**Figure 1:**
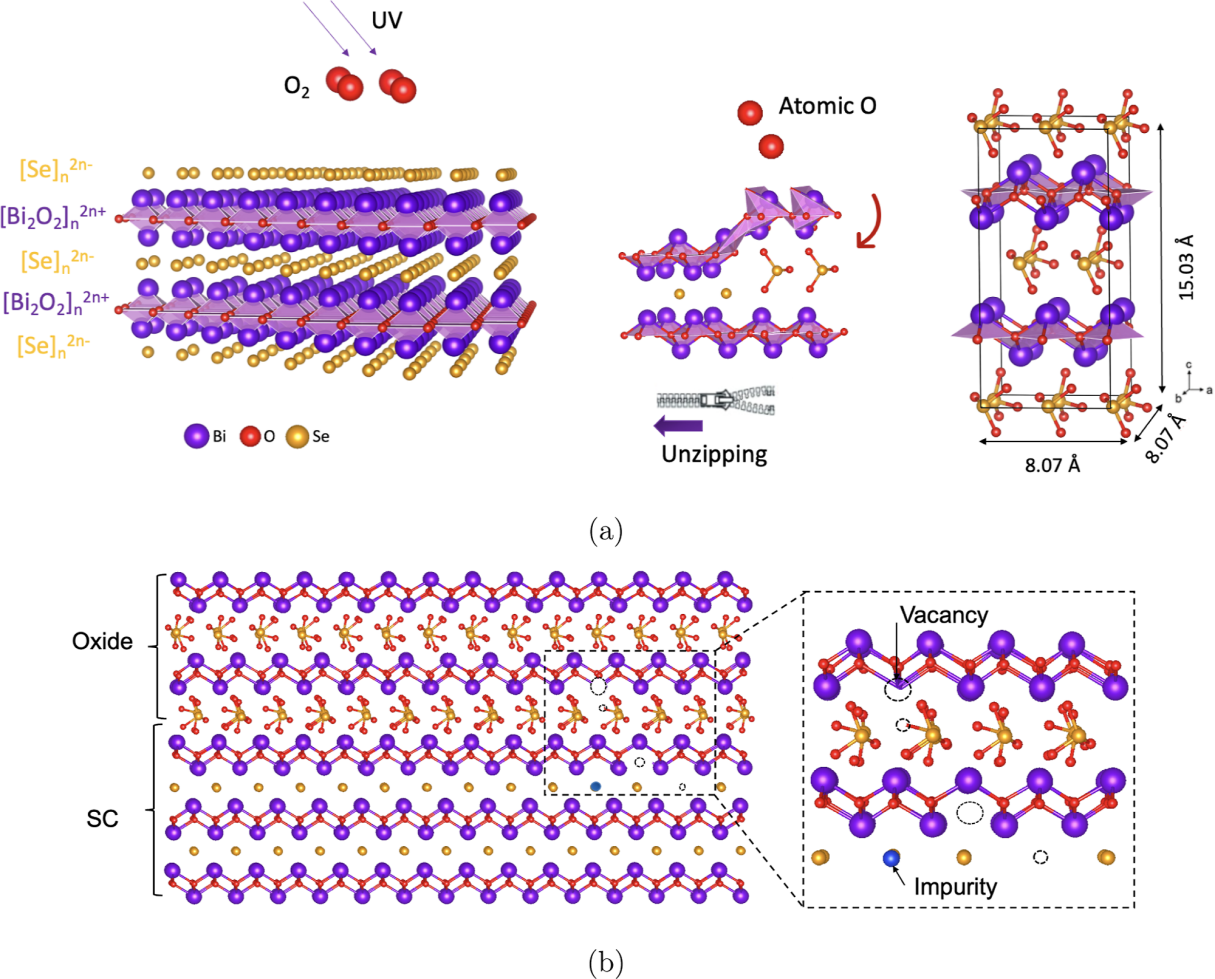
(a) From left to right: crystal structure of 2D Bi_2_O_2_Se with alternating ${\left[{Bi}_{2}O_{2}\right]}_{n}^{2+}$ and [Se]_*n*_^2–^ layers; a schematic of the UV-assisted intercalative oxidation process preserving the [Bi_2_O_2_] framework and enlarging the interlayer spacing; and the crystal structure of the resulting intercalative oxide β-Bi_2_SeO_5_. (b) The resulting smooth and dangling-bond-free Bi_2_O_2_Se/Bi_2_SeO_5_ heterostructure including a few exemplary point defects, such as two oxygen vacancies.

Despite the significant progress in synthesizing and characterizing the Bi_2_O_2_Se/β-Bi_2_SeO_5_ heterostructure, the presence and role of intrinsic defects within the native oxide, and their impact on device reliability, remain largely unexplored. Understanding the behavior of native point defects is essential for the successful application of any semiconductor, as defects typically control electrical and optical properties. Atomic defects in the oxide, especially at or near the interface with Bi_2_O_2_Se as illustrated in Figure 1b, can act as charge carrier trapping sites. This charge trapping at oxide defects fundamentally impacts the stability and reliability of transistors. For example, in small-area devices, charge trapping leads to phenomena like random telegraph noise (RTN),^26^ which arises from single charge capture or emission events at individual defects. These charge transitions are observed as stochastic discrete steps in the drain current (*I*_D_) at constant gate voltage (*V*_G_), which alter device electrostatics and cause undesired threshold voltage shifts (Δ*V*_th_). In large-area devices, numerous trapping events collectively cause smooth Δ*V*_th_ shifts at high temperatures and gate biases, known as bias temperature instability (BTI),^26^ a key reliability issue. Both BTI and RTN stem from oxide defects and can be modeled within the same theoretical framework,^26,27^ which underscores the importance of understanding the nature and energetic properties of intrinsic defects. Another frequently observed reliability issue in 2D-FETs is the hysteresis in the transfer characteristics, which is observed as a difference in voltage shifts (between consecutive *I*_D_(*V*_G_) up and down sweeps) and is quantified by the hysteresis width. This phenomenon is also primarily caused by charge trapping at both interface and gate oxide traps.^28^

Intrinsic point defects such as oxygen vacancies (V_O_) and selenium vacancies (V_Se_) are expected to exist in significant concentrations in the Bi_2_O_2_Se structure.^29^ In addition, β-Bi_2_SeO_5_ contains oxygen atoms in two distinct bonding environments, including those within the preserved [Bi_2_O_2_] framework and those in the interlayer [SeO_3_] tetrahedra. Hence, a variety of oxygen and selenium vacancies are expected.

In this work, we investigate intrinsic defects in Bi_2_O_2_Se and its native insulator, β-Bi_2_SeO_5_, to systematically examine defect formation within these materials. By employing density functional theory (DFT), we identify the key defect parameters that govern nonradiative multiphonon (NMP) charge-transfer processes,^30,31^ including formation energies, thermodynamic trap levels (expressed as charge-state transition levels), and relaxation energies.^27,32^ We then validate these insights by correlating them with experimental hysteresis and BTI device measurements from a prototype Bi_2_O_2_Se/Bi_2_SeO_5_ FET. Applying these models enables the prediction of device degradation and the analysis of reliability issues.^33^ This work focuses specifically on intrinsic defects within the β-Bi_2_SeO_5_ layer, such as oxygen and selenium vacancies. Addressing the previously recognized lack of a comprehensive understanding of intrinsic defects in the Bi_2_O_2_Se/β-Bi_2_SeO_5_ heterostructure, this study presents a systematic DFT analysis of defect formation within the oxide layer. A comprehensive insight into these defects by DFT-derived quantities such as trap levels and relaxation energies is critical for identifying the origins of stability and reliability issues as well as for guiding the optimization of material quality and device reliability in Bi_2_O_2_Se/β-Bi_2_SeO_5_ heterostructures. Thus, these results may help improve the stability and reliability of Bi_2_O_2_Se/β-Bi_2_SeO_5_-based nanoelectronic devices.

## Results and Discussion

### Electronic Properties of Point Defects

In this section, we first investigate the structure and electronic properties of Bi_2_O_2_Se and its native oxide, followed by an analysis of intrinsic defects in the material and a subsequent discussion of defects in the native oxide. In the next step, the formation energies, trap levels, and relaxation energies within the oxide are examined. Details about the computational setup are given in the Methods Section. Finally, the impact of these defects on the reliability of a prototype device under investigation was analyzed.

### Bismuth Oxyselenide Bi_2_O_2_Se (Semiconductor)

Our DFT calculations predict that bulk Bi_2_O_2_Se has an indirect bandgap of approximately 1.09 eV in its bulk form (see Figure 2b). Bi_2_O_2_Se possesses a tunable bandgap (approximately 0.8–1.9 eV)^19,34^ that depends on its thickness, making it suitable for optical applications across a range of wavelengths. This theoretical value is in reasonable agreement with experimental results of bulk Bi_2_O_2_Se, where angle-resolved photoemission spectroscopy measurements reveal a slightly smaller bandgap of about 0.8 eV.^17^ The difference can be attributed to the neglect of spin–orbit coupling in the present calculations. Notably, Bi_2_O_2_Se does not exhibit electrically active surface states within its bandgap, even when the characteristic half-occupied surfaces featuring selenium vacancy dimers (as shown in Figure 2a) are present.^35,36^ This contrasts with the typical behavior of vdW materials, which possess a vdW gap and exhibit comparatively weak interlayer binding energies. The projected density of states (PDoS) analysis (see Figure 2a) further indicates that the conduction-band minimum (CBM) is predominantly derived from Bi orbitals, in particular, the Bi p-orbital. In contrast, the valence-band maximum (VBM) mainly arises from the p-orbitals of Se, with notable contributions from oxygen atoms within the Bi_2_O_2_Se framework, in agreement with previous findings.^22,37^

**Figure 2:**
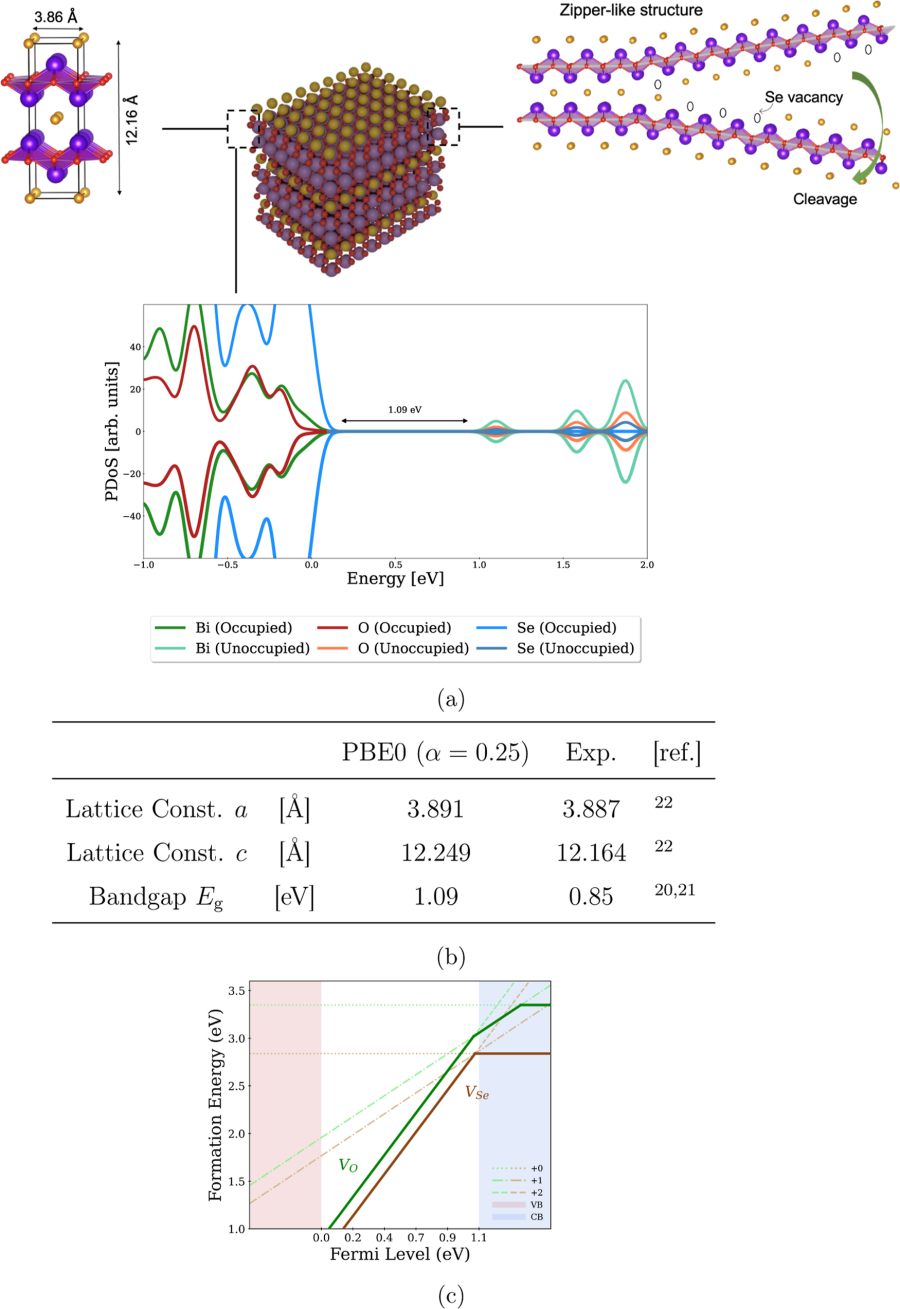
Atomic structure of the layered zipper material Bi_2_O_2_Se, including (a) the structure and the PDoS, which indicate a bandgap of 1.09 eV; (b) a comparison of Bi_2_O_2_Se bulk properties calculated using our DFT setup with available experimental data; and (c) the formation energy of oxygen and selenium vacancy defects in Bi_2_O_2_Se as a function of the Fermi level.

Until now, Bi_2_O_2_Se-based FETs have mostly exhibited n-type characteristics, with their channels typically remaining conductive even at zero gate voltage. This unintentional n-type doping is often attributed to the presence of native point defects.^29,38^ To provide a more comprehensive microscopic understanding of this n-type behavior, this study employs DFT to not only investigate intrinsic defect properties by thoroughly examining their various possible charge states and corresponding formation energies but also to analyze their charge-transition dynamics. The lattice parameters of the relaxed supercell are *a* = 19.45 Å, *b* = 19.45 Å, *c* = 24.49 Å.

#### Vacancies in Bi_2_O_2_Se

Defects are a fundamental aspect of electronic and thermoelectric properties of Bi_2_O_2_Se, as they significantly affect its charge carrier concentration and overall performance.^20,38^ All oxygen atoms in Bi_2_O_2_Se are located in the [Bi_2_O_2_]^+^ layer and coordinated to four bismuth atoms through strong covalent bonds. Introducing an oxygen vacancy by removing an O atom (V_O_) from this layer leaves four neighboring Bi atoms undercoordinated. In the neutral charge state (V_O_^0^), the associated defect-derived state lies approximately 0.58 eV above the VBM and is fully occupied by two electrons. For the singly positive charge state (V_O_^+1^), the occupied spin-up state is located at approximately 1.05 eV above the VBM, while the corresponding unoccupied spin-down state appears close to the conduction-band minimum, at approximately 1.08 eV above the VBM. For the doubly positive charge state (V_O_^+2^), the vacancy-related state becomes fully unoccupied and also appears close to the conduction-band minimum, at approximately 1.07 eV above the VBM. The close energetic proximity of these unoccupied states to the CBM indicates shallow donor-like behavior rather than the formation of deep localized trap states. The PDoS for each charge state is presented in Figure S1b in the Supporting Information. Removal of an oxygen atom introduces a vacancy-derived state with enhanced weight on the neighboring Bi atoms in the Bi_2_O_2_ layer. This defect-derived state arises from the hybridization of Bi orbitals surrounding the vacant site, while the unoccupied states in the positive charge states lie close to the conduction-band edge. This energetic proximity to the conduction band indicates that oxygen vacancies behave as shallow donors and can readily contribute free electrons to the system. The formation energy of the oxygen vacancy as a function of the Fermi level is shown in Figure 2c. The neutral V_O_ and singly charged (V_O_^+1^) configurations are higher in energy than the doubly charged state V_O_^+2^ for almost all Fermi levels within the bandgap. This indicates that the oxygen vacancy is a double donor. This result, confirming V_O_ acts as a shallow donor defect generating free carriers, aligns with prior theoretical work, which concluded that the defect transition levels in Bi_2_O_2_Se are located above the CBM.^39^

In addition to oxygen vacancies, selenium vacancies (V_Se_) are recognized as crucial and prominent native point defects in Bi_2_O_2_Se.^29^ The formation of V_Se_ in the Bi_2_O_2_Se structure is energetically competitive under the chemical-potential conditions considered (see Figure 2c) and is favored under Se-poor growth conditions. This is because selenium atoms occupy a unique plane within the material’s structure, forming [Se]^2*n*−^ layers that are weakly bonded by electrostatic interactions to the neighboring [Bi_2_O_2_]^2*n*+^ layers. These V_Se_ bulk defects act as shallow donors. A crucial characteristic that directly aids n-doping is that their donor levels lie energetically above the conduction-band bottom in energy and they do not introduce any in-gap states in any charge state. While exfoliated Bi_2_O_2_Se surfaces exhibit a zipper-like structure with 50% selenium coverage and patterned surface Se vacancies forming a 2 × *n* reconstruction,^21,36^ these surface vacancies likewise act as shallow donors, consistent with the behavior of bulk Se vacancies. This unique characteristic leads to the spontaneous ionization of free electrons from these donor sites without requiring thermal activation or overcoming an activation barrier, a phenomenon termed self-modulation doping.^29^ Each V_Se_ site is capable of donating two electrons to the crystal, contributing to the unintentional n-type conductivity commonly observed in Bi_2_O_2_Se. The exceptionally high electron mobility observed in Bi_2_O_2_Se is fundamentally driven by this unique intrinsic mechanism of self-modulation doping. A key aspect is the spatial separation between conducting electrons and their ionized donor sites. The wave function of the lowest conduction-band state is predominantly located in the Bi_2_O_2_ layers, primarily derived from Bi orbitals, while donor defect states from V_Se_ reside in the Se layers. This intrinsic separation strongly suppresses scattering caused by ionized donor sites, thereby significantly contributing to the large electron mobility observed in Bi_2_O_2_Se, especially at low temperatures. The importance of this mechanism has been previously identified as a defining feature of Bi_2_O_2_Se.^29^ Our hybrid functional calculations confirm this spatial separation, showing that V_Se_ introduce defect states above the conduction band while the CBM remains dominated by Bi orbitals within the Bi_2_O_2_ layers. The shallow donor levels from the Se vacancies, along with those associated with oxygen vacancies, are believed to contribute to the intrinsic n-type conductivity commonly observed in Bi_2_O_2_Se.^29^ Because these donor levels are resonant with or above the conduction-band minimum, they are expected to ionize spontaneously rather than requiring thermal activation, thereby enhancing the free-electron density. The formation of both V_Se_ and V_O_ is highly dependent on the thermodynamic growth conditions, specifically the synthesis temperature and the elemental chemical potentials (e.g., Se concentration). Formation energy calculations indicate that selenium vacancies have a low formation energy (Figure 2c), comparable to that of oxygen vacancies and sensitive to the growth conditions, suggesting that they exist in considerable concentrations in Bi_2_O_2_Se. Synthesizing under more Se-poor conditions promotes the formation of V_Se_, leading to a higher electron mobility as confirmed by electrical measurements.^29^ Conversely, Se-rich conditions suppress donor-type V_Se_ formation, favoring acceptor-type bismuth vacancies (V_Bi_) and interstitial selenium atoms (Se_in_), which can lead to more insulating behavior. However, our analysis predominantly focuses on donor-like defects in the channel, as these support the observed n-type operation.^29^

### Bismuth Oxoselenate β-Bi_2_SeO_5_ (Dielectric)

We relaxed the initial β-Bi_2_SeO_5_ lattice obtained from Khakbaz et al.^22^ with our DFT setup, resulting in optimized lattice parameters of *a* = *b* = 8.23 Å and *c* = 15.50 Å. Furthermore, our calculations predict a wide bandgap of approximately 3.5 eV, in good agreement with recent reports of around 3.7 eV.^22^ The valence band is mainly composed of oxygen states from the [Bi_2_O_2_]^2+^ layers, and the conduction band is primarily composed of Bi states, with some contribution from Se states. Additionally, β-Bi_2_SeO_5_ exhibits anisotropic and relatively large effective electron masses compared to Bi_2_O_2_Se, which is advantageous for insulating applications since this leads to a reduced tunneling probability.^22^ For defect and impurity calculations, we construct a 2 × 2 × 1 supercell comprising 256 atoms by repeating the primitive cell of β-Bi_2_SeO_5_ from which the initial structure was derived^22^ (see Figure 1a). This supercell is large enough for accurately studying defect properties, as it ensures that the charged defects are sufficiently separated from their periodic images to minimize artificial electrostatic interactions.

#### Oxygen-Related Point Defects

In β-Bi_2_SeO_5_, oxygen atoms are present in two distinct bonding environments: those within the Bi_2_O_2_ framework and those in the interlayer SeO_3_ tetrahedra. Each of the three oxygen atoms within the SeO_3_ tetrahedra is bonded to one Se atom. Therefore, forming an oxygen vacancy (V_O_) breaks only one of the Se–O bonds, while the other two remain intact, leaving the selenium atom partially bonded. This results in a localized defect state primarily composed of O 2p orbitals, with minor variations depending on which of the three oxygen atoms is removed. The PDoS for the oxygen vacancy in different charge states is illustrated in Figure 3a. In the neutral charge state V_O_^0^, the defect state is located 1.29 eV above the VBM and is fully occupied with two electrons of opposite spins. In the singly positive charge state, V_O_^+1^, the defect state is occupied by one electron, the occupied spin-up state is located approximately 0.53 eV above the VBM, while the spin-down state remains unoccupied and lies deep within the bandgap, around 2.7 eV above the VBM. In the doubly positive charge state, V_O_^+2^, the defect state is at 3.3 eV above the valence band and unoccupied. In V_O_^+2^, instead of an isolated vacancy, the system undergoes structural relaxation in which a neighboring oxygen atom shifts toward the vacant site as shown in Figure 3a (right). This results in an oxygen atom being shared between two adjacent Se atoms. Such a defect configuration is commonly known as a split vacancy, where the missing O site is partially reoccupied by a neighboring O atom, creating a bridging configuration. The formation energy of the oxygen vacancy as a function of the Fermi level is shown in Figure 3d. For Fermi levels below the +1/+ 2 charge-transition level, the doubly ionized vacancy V_O_^+2^ is the lowest-energy configuration. Above the +1/0 charge-transition level (CTL), the neutral vacancy V_O_^0^ becomes the most stable state. In comparison, the neutral V_O_^0^ and singly ionized V_O_^+1^ configurations are higher in energy than the doubly ionized V_O_^+2^ over much of the Fermi-level range, while the neutral state becomes stable for Fermi levels above roughly the midgap of β-Bi_2_SeO_5_ (Figure 3d), indicating that the oxygen vacancy within the SeO_3_ tetrahedra behaves as a deep hole trap.

**Figure 3:**
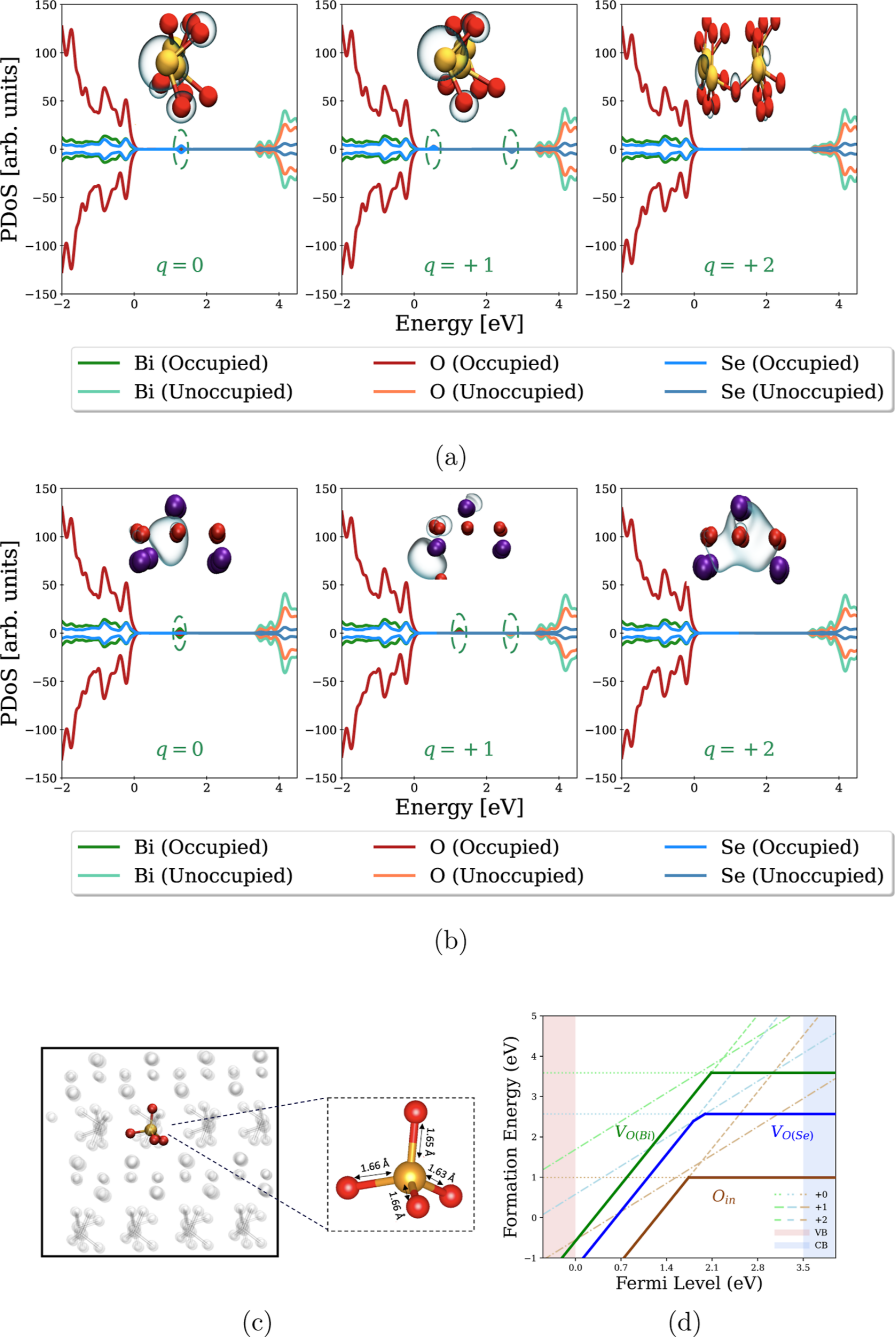
(a) PDoS for an oxygen vacancy (V_O_) bonded to a Se atom in charge states *q* = 0, +1, +2. (b) PDoS for an oxygen vacancy located in the Bi_2_O_2_ layer, also for *q* = 0, +1, +2. Insets in (a) and (b) show the corresponding HOMO isosurfaces (blue) at 0.05 e/Å^3^; within each panel, the subimages (left–right) correspond to the neutral, singly positive, and doubly positive charge states. (c) Structural configuration of the relaxed oxygen interstitial defect in Bi_2_SeO_5_. (d) Formation energy as a function of the Fermi level for the corresponding defect in different charge states.

The other oxygen atoms in the β-Bi_2_SeO_5_ structure reside within the planar layers composed of bismuth and oxygen, which form the positively charged ${\left[Bi_{2}O_{2}\right]}_{n}^{2n+}$ frameworks, as illustrated in Figure 1a. These layers are derived from the parent Bi_2_O_2_Se structure and are characterized by strong covalent bonding. Compared to the oxygen atoms in the [SeO_3_] tetrahedra, those in the [Bi_2_O_2_] framework exhibit significantly smaller dynamic displacements,^22^ indicating higher lattice stability. Each oxygen atom in the [Bi_2_O_2_] layer is bonded to four bismuth atoms, so the formation of an oxygen vacancy in this region would involve breaking strong covalent bonds and result in the creation of four dangling bonds. The PDoS for the oxygen vacancy in different charge states is illustrated in Figure 3b. In the neutral charge state V_O_^0^, the defect state appears 1.3 eV above the VBM and is fully occupied with two electrons of opposite spins and as depicted in Figure 3b (left) the defect state is highly localized around the vacancy site. Upon removal of one electron, V_O_^+1^ forms, resulting in one occupied and one unoccupied defect state of opposite spins. The occupied state is located approximately 1.3 eV above the VBM and remains localized (Figure 3b (middle)), while the unoccupied state lies deep within the bandgap, around 2.7 eV above the valence band. In the doubly positive charge state, V_O_^+2^, the defect states are unoccupied and lie 3.31 eV above the valence band, closely below the CBM. The formation energy of the oxygen vacancy as a function of the Fermi level is shown in Figure 3d. The formation energy of oxygen vacancies in the [Bi_2_O_2_] layer is higher than that of oxygen atoms bonded to Se, presumably due to the stronger covalent bonding within the layer. The neutral V_O_^0^ and singly ionized V_O_^+1^ configurations are higher in energy than the doubly ionized charge state V_O_^+2^ for Fermi levels below approximately 2.1 eV. This indicates that also the oxygen vacancy within the [Bi_2_O_2_]_*n*_^2*n*+^ frameworks is a deep hole trap, accepting up to two holes.

Due to the unique UV-assisted intercalative oxidation process by which this native oxide is formed, the formation of oxygen interstitials (O_in_) in the β-Bi_2_SeO_5_ structure is highly plausible. In the pristine structure, selenium atoms are coordinated by three oxygen atoms, forming SeO_3_ tetrahedra units characteristic of a +4 oxidation state. To investigate the impact of oxygen interstitials on the structure of β-Bi_2_SeO_5_, a single oxygen atom was inserted into the lattice near a selenium site, followed by structural relaxation. Upon relaxation, the interstitial oxygen atom migrated toward a nearby SeO_3_ unit and formed a bond with the selenium atom. The resulting Se–O bond lengths range from 1.63 Å to 1.66  Å, and the coordination geometry transitions toward a distorted tetrahedral configuration (see Figure 3c). This structural modification effectively creates a SeO_4_-like unit embedded within the Bi_2_SeO_5_ lattice. The additional Se–O bond introduces significant local distortion and suggests a partial oxidation of the selenium center from Se^4+^ toward Se^6+^. This partial oxidation raises the structural possibility that the native oxide interlayer may locally deviate from the conventionally reported Bi_2_SeO_5_ composition toward an oxygen-richer Bi_2_SeO_6_-like environment, although further investigations are required to clarify the actual composition. The formation energy of the oxygen interstitial as a function of the Fermi level is shown in Figure 3d.

#### Selenium Vacancies

Following our earlier discussion of selenium vacancies (V_Se_) as native shallow donor defects in the layered Bi_2_O_2_Se semiconductor, we find that their behavior differs significantly in the native oxide, β-Bi_2_SeO_5_. In the oxide, the formation of a selenium vacancy results in the three oxygen atoms previously bonded to Se becoming unbonded. After atomic relaxation, two of these oxygen atoms pair to form a neutral O_2_ molecule, while the third remains unbonded within the layer (see Figure 4a). This configuration minimizes the system’s energy by passivating the resulting dangling bonds through the O–O bond formation. The PDoS analysis in Figure S2 (left) in the Supporting Information shows the emergence of a localized defect state above the valence band, predominantly composed of oxygen 2p orbitals, indicating that the electronic structure near the vacancy is strongly influenced by oxygen. This defect state corresponds to the HOMO of a neutral O_2_ molecule; although molecular in origin, its energy lies within the bandgap, enabling such interstitial O_2_ species to act as charge-trapping centers in this material. Visualization of the HOMO confirms that two of the three oxygen atoms previously coordinated to the removed selenium form a neutral O_2_ molecule (see Figure 4b). Based on these evaluations, the formation of a selenium vacancy appears unlikely.

**Figure 4:**
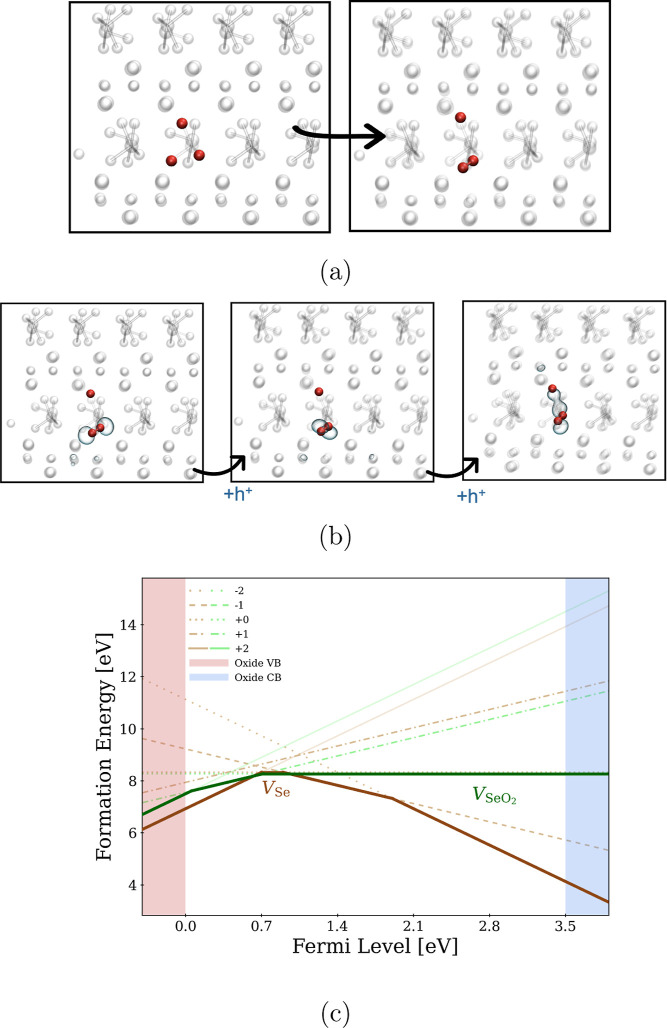
(a) Structural relaxation following the introduction of a Se vacancy. The relaxed structure shows the formation of an O_2_ molecule. (b) HOMO for the Se vacancy in three different charge states with isosurfaces (blue) at 0.05 e/Å^3^. (c) Formation energy as a function of the Fermi level for Se and SeO_2_ vacancies in different charge states.

During the oxidation process, we assume that a single O is captured in the void, effectively leading to a $V_{SeO_{2}}$ vacancy complex. Following structural relaxation, nearby atoms, particularly the bismuth atoms, undergo displacement. The remaining oxygen atom forms new bonds with two adjacent bismuth atoms, with bond lengths of approximately 2.14 Å and 2.34 Å. This rearrangement leads to local structural distortion and overcoordination of the Bi atoms. Effectively, this defect configuration creates an electronic state located close to the conduction band that is primarily composed of O 2p and Bi 6p orbitals (Figure S2, right). While selenium vacancies can trap electrons, the trapped charge preferentially localizes on the O_2_ unit formed during relaxation rather than on the remaining single oxygen atom. Figure 4c depicts the formation energy diagrams of the Se vacancy (V_Se_) and (V_SeO_2__) vacancy as a function of the Fermi level across multiple charge states. The resulting defect structure of the SeO_2_ vacancy induces a comparable electronic perturbation to that of a single Se vacancy. This similarity arises from the fact that both defects are centered on the Se site and lead to analogous local structural relaxations and electronic hybridizations, particularly involving nearby Bi atoms.

#### Charge-Transition Levels and Relaxation Energies

The CTL, also referred to as the trap level E_T_ in the literature, corresponds to the Fermi level E_F_ at which the formation energies of a defect in two distinct charge states are equal. Therefore, the CTL marks the thermodynamic crossover between stability regions and determines the defect’s preferred charge state with respect to the Fermi level. The CTLs can be directly derived from the formation energy. When a defect traps a charge, the localized electron within the oxide disrupts the device’s electrostatics, leading to changes in critical device parameters such as the threshold voltage.^40,41^ The CTL is the only defect parameter directly measurable through electrical experiments. In an MOS device, application of a gate bias shifts the trap level due to band bending near the interface and the electric field in the oxide. When the Fermi level matches the CTL, the two charge states involved become equally stable, leading to equal occupancy and maximum power of the RTN signal.^42^ The CTL of an oxide defect is an intrinsic property of the host material and generally independent of the electronic bands of the substrate. However, the morphology of the interface to the substrate can indirectly influence the CTL through factors like strain or changes in stoichiometry. The CTLs were calculated for all possible transitions of the point defects in β-Bi_2_SeO_5_ and are presented below for various defect candidates within the bandgap of Bi_2_SeO_5_ (see Figure 5a). Our analysis primarily concentrates on transitions involving the transfer of a single electron or hole.

**Figure 5:**
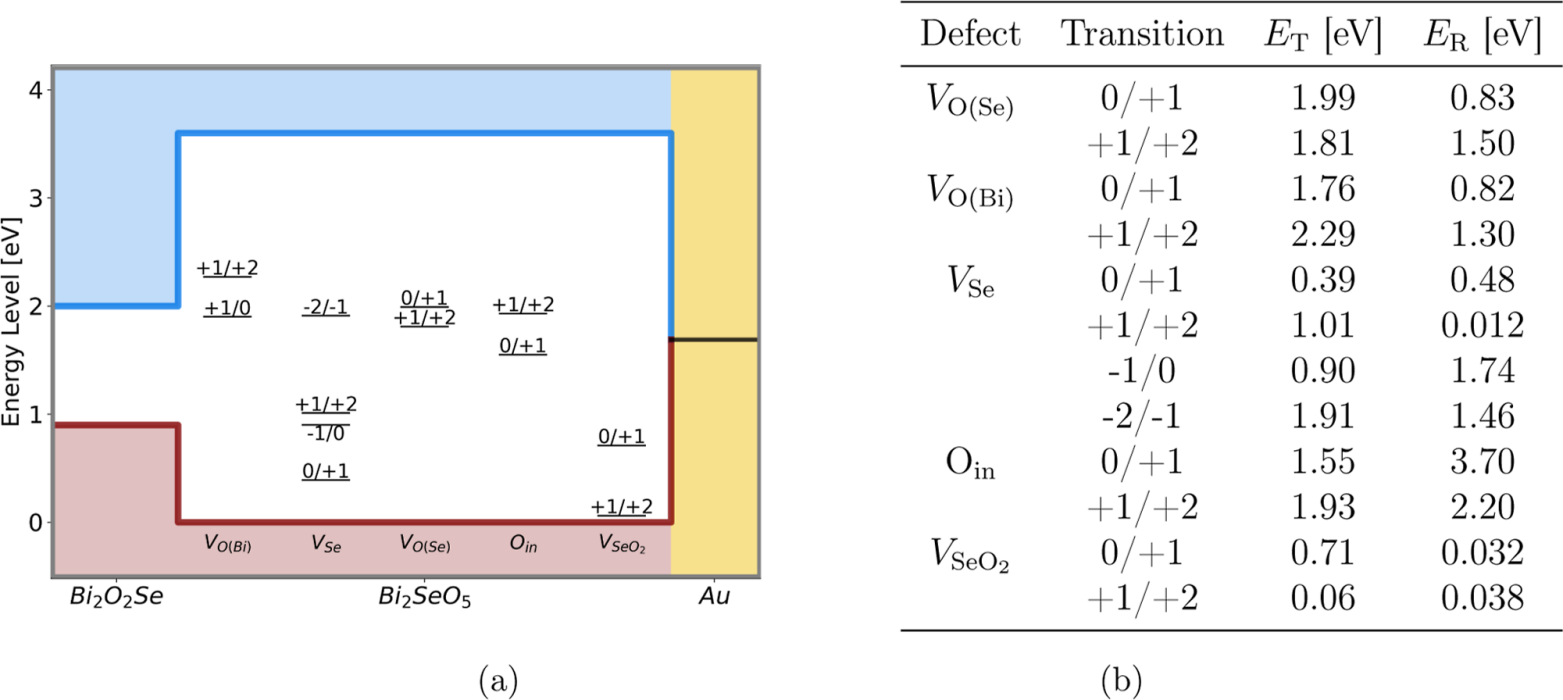
(a) Trap levels of different defects within the bandgap of Bi_2_SeO_5_ together with the band edges of Bi_2_O_2_Se. (b) Theoretical defect transitions and parameters predicted with DFT. *E*_T_ is given relative to the Bi_2_SeO_5_ valence-band maximum.

In addition to the CTL, the relaxation energies *E*_R_ provide insights into the potential energy surfaces of defects in different charge states, as they are related to the curvature near the energy minima. Within the harmonic approximation, the crossing point and classical barrier between potential energy curves of different charge states are fully defined by the CTL and the relaxation energies.^27^ Therefore, *E*_R_ strongly influences the defect’s capture and emission time constants.^26^ For a charge transition from 0 to −1 (or +1), the relaxation energy is denoted *E*_Relax_^0/–^ (*E*_Relax_^0/+^), and vice versa for *E*_Relax_^–/0^ (*E*_Relax_^+/0^). These energies are calculated using single-point calculations of defects in relaxed geometries of the opposite charge states. For instance, the relaxation energy for the 0/– transition is given by

3 $$E_{relax}^{0/-}=V^{-}\left(Q_{1}\right)-V^{-}\left(Q_{2}\right)$$

where *V* ^–^ is the potential energy of the negatively charged defect at the equilibrium configurations *Q*_1_ and *Q*_2_ of the defect in charge states 0 and −, respectively. Thus, *E*_Relax_ can also be interpreted as the energy released to the lattice after a nonradiative charge-transfer event. In the case of nonradiative transitions, the relaxation energies often dominate the classical energy barrier for charge transfer. Relaxation energies for each defect type are presented in Figure 5b. The charge-transition levels shown in Figure 5a and the corresponding relaxation energies listed in Figure 5b provide insight into which defects are expected to be relevant for device reliability.

### Impact of Defects on Device Reliability

To comprehensively evaluate the impact of intrinsic and interstitial point defects on device reliability, we integrate experimental measurements with TCAD simulations to study a device based on a Bi_2_O_2_Se/Bi_2_SeO_5_ heterostructure. Specifically, we examine two prevalent reliability concerns in 2D-FETs: BTI and hysteresis. Both phenomena are predominantly associated with charge trapping within the gate insulator and at the semiconductor–dielectric interface;^42^ however, counterclockwise hysteresis or anomalous BTI can also originate from the movement of mobile ions in the dielectric.^43,44^ The prototype device investigated is a gate-all-around (GAA) FET, an architectural innovation considered the latest and most advanced device sample with this material system.^45^ Structurally, this device involves the Bi_2_O_2_Se functioning as the channel core, which is entirely wrapped by the layered, Bi_2_SeO_5_, acting as the shell. This arrangement forms a core–shell GAA structure, where the Bi_2_SeO_5_ layer is epitaxially integrated onto the Bi_2_O_2_Se surface, forming an atomically smooth, lattice-matched interface because the semiconductor shares the Bi–O layer with the oxide. The device was grown vertically on mica substrates using CVD and was subjected to ultraviolet (UV)-assisted intercalative oxidation before being subsequently physically transferred onto Si/SiO_2_ substrates. This sequence was necessary to enable oxide growth on the bottom side of the vertically grown nanostructure, achieving the fully wrapped core–shell structure. The structural details are revealed in the cross-sectional high-angle annular dark-field scanning transmission electron microscopy images shown in Figure 6a. Further details on the fabrication processes can be found in previous works.^45^ The Bi_2_O_2_Se devices exhibit clear n-type behavior, while the negative *V*_th_ suggests a significant amount of positive charge next to the channel, as can be seen in Figure 6b.

**Figure 6:**
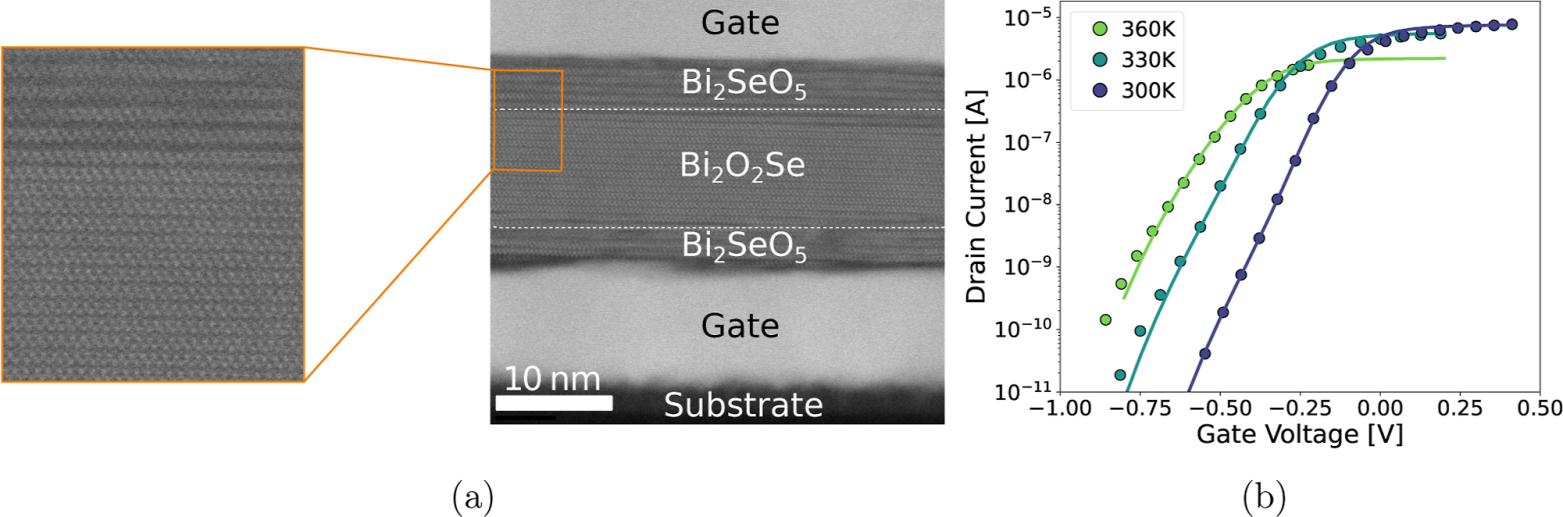
(a) The GAA-FET at two different magnifications showing the Bi_2_SeO_5_/Bi_2_O_2_Se/Bi_2_SeO_5_ stack surrounded by the metallic gate contact (the light regions). (b) The measured *I*_D_ over *V*_G_ curve with *W* = 3 μm, *L* = 3.2 μm, showing good agreement of the model (lines) with the measurement data (circles) and an *I*_on_ of 1.5 μA/μm.

### Bias Temperature Instability and Hysteresis

BTI is defined as a shift in the threshold voltage (Δ*V*_th_) following the application of bias and is strongly accelerated by temperature.^46^ This shift poses a serious concern for device reliability, as it alters the point on the transfer characteristic at which the device turns on. This threshold voltage shift is primarily attributed to charge trapping within the gate oxide or the interfacial layer of the device.^47,48^ Therefore, accurately predicting BTI degradation over a device’s operational lifetime requires a detailed understanding of the distribution of active defects and their charge trapping behavior under various temperatures and gate biases. To investigate the dynamics of defect-related charge trapping events, we performed PBTI measurements using a stress-relaxation sequence.

First, the device was subjected to a stress phase, during which a constant positive gate voltage, *V*_G,stress_, equal to the maximum gate voltage of the corresponding range used in the *I*_D_(*V*_G_) measurements, was applied for fixed durations (*t*_stress_ = 1, 10, and 100 s). After the stress phase, *I*_D_(*V*_G_) measurements were performed immediately after stress and subsequently after logarithmically spaced relaxation intervals. From these measurements, the *V*_th_ shift was extracted by using a constant-current criterion, typically at the drain current corresponding to the threshold voltage (*V*_th_). During the relaxation phase, the gate voltage was set to *V*_G,relax_, equal to the minimum gate voltage of the corresponding range, while the drain voltage was set to *V*_D_ = 0.1 V during the *I*_D_(*V*_G_) measurements and kept at 0 V during the stress phase and relaxation intervals without measurements. The resulting device degradation induced by the applied electrical stress and the subsequent recovery are shown for three different temperatures in Figure 7b–d.

**Figure 7:**
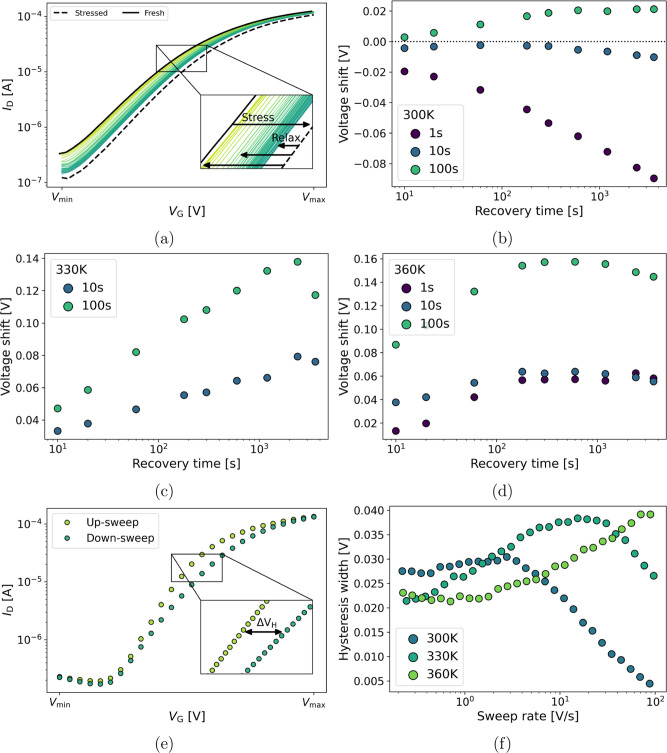
(a) Schematic BTI Δ*V*_th_ extraction method, starting with a fresh device, a *V*_G_ stress phase of duration *t*_stress_ followed by a recovery phase. *I*_D_(*V*_G_) curves are measured to track the time evolution of *V*_th_. Measured PBTI shift at (b) 300 K, (c) 330 K, and (d) 360 K with respect to the baselines (measured *V*_th_ before the recovery phase). (e) Schematic hysteresis extraction method, where Δ*V*_H_ is the difference between the up and the down sweep *V*_th_. (f) Evaluated hysteresis as a function of sweep rate at three temperatures.

In addition to BTI, another closely linked reliability issue is hysteresis, which is expressed as a shift in the threshold voltage between forward (ramp up) and reverse (ramp down) gate voltage sweeps in the transfer characteristics. Hysteresis, similar to BTI, constitutes a major stability concern in 2D FETs and is primarily attributed to charge trapping at interface states and within the gate oxide. To determine whether the previously identified defects could be responsible for hysteresis in transistors with a Bi_2_SeO_5_ gate dielectric, we performed double sweep *I*_D_(*V*_G_) measurements at various sweep rates on the device. This approach enables differentiation between fast and slow trapping processes by examining the dependence of the hysteresis width, Δ*V*_H_, on the sweep rate and temperature. The hysteresis width is extracted using the very same constant current criterion as for the BTI experiments described above, thereby providing a quantitative measure of defect-related trapping dynamics (see Figure 7e). Figure 7f presents the hysteresis response of the device under different sweep rates and at different temperatures. The observed hysteresis exhibits a clockwise (CW) behavior at all three measured temperatures, which confirms charge trapping as the main reliability problem.^44^

Furthermore, for both the BTI and hysteresis investigations, the measurement procedure started with a 1 min preconditioning phase, during which the gate voltage was swept over the same temperature-dependent range used for the subsequent measurement at *V*_D_ = 0.1 V and a sampling frequency of 100 Hz. This was followed by an *I*_D_(*V*_G_) sweep to determine the corresponding gate-voltage range, adapted to the respective *V*_th_. This approach, described in ref 44, should minimize the effects of prior measurements and temperature-related drift.

We employed the effective single defect decomposition (ESiD) method^33,47^ to extract a physically consistent distribution of traps capable of reproducing the experimentally observed PBTI and hysteresis results. As shown in Figure 8a–c, the simulated BTI degradation agrees very well with the experimental data measured at three different temperatures. Further, the simulated hysteresis shows excellent agreement with the experimental data measured at three different temperatures (see Figure 8d).

**Figure 8:**
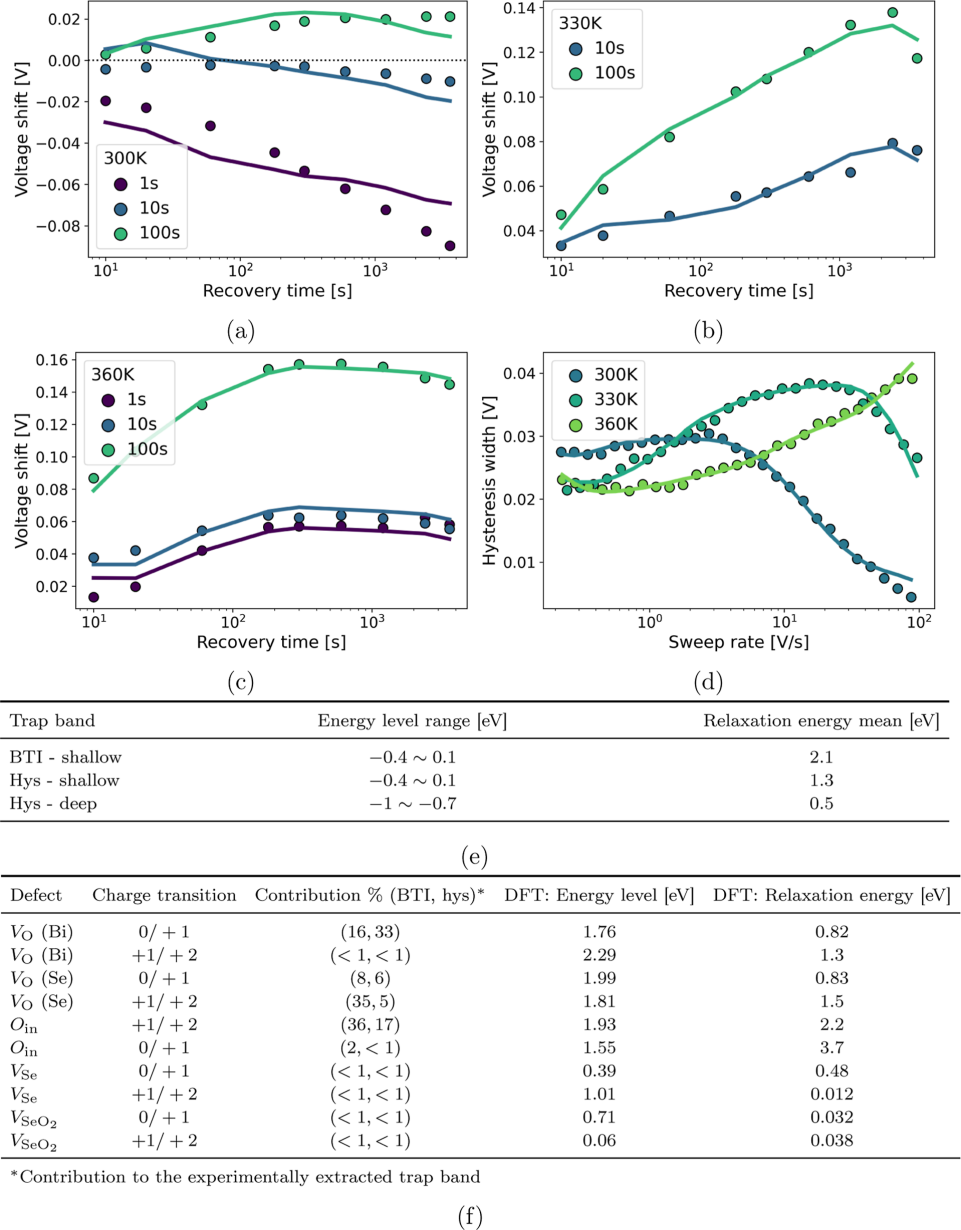
Simulated (lines) and measured (circles) BTI shifts at (a) 300 K, (b) 330 K, and (c) 360 K. (d) Simulated (lines) and measured (circles) at three different temperatures. (e) Extracted trap band characteristics obtained from BTI and hysteresis data. (f) The relevant structural point defects matching the extracted trap bands from reliability measurements, dominating hysteresis and BTI. Gray-shaded rows indicate defects showing the best agreement between DFT predictions and ESiD-extracted trap bands.

As a next step, we compared the experimentally derived defect parameters with the point defects previously analyzed by DFT. Figure 8f compares the charge-transition levels (CTLs) and relaxation energies of the DFT-predicted point defects with the experimentally extracted distributions of trap levels (*E*_T_) and relaxation energies (*E*_R_) obtained by device simulations (ESiD). These comparisons indicate that deep oxygen-related defects in β-Bi_2_SeO_5_, including oxygen vacancies in the [SeO_3_] tetrahedra and the [Bi_2_O_2_] layers (VtnqethylsafeO (Bi), VtnqethylsafeO (Se)) as well as oxygen interstitials O_in_, act as the primary charge-capturing centers responsible for BTI and hysteresis.

## Conclusions

This work comprehensively investigates intrinsic point defects in the high-mobility layered semiconductor Bi_2_O_2_Se and its high-κ native oxide, β-Bi_2_SeO_5_, using a combination of first-principles calculations, device simulations, and experimental characterization. Our thermodynamic CTL analysis suggests that both V_Se_ and V_O_ act as shallow donors, explaining the commonly observed natural n-type conductivity in Bi_2_O_2_Se-based FETs. More importantly, in the native dielectric β-Bi_2_SeO_5_, our theoretical and experimental analyses converged to demonstrate that oxygen vacancies (including those within both the [SeO_3_] tetrahedra and [Bi_2_O_2_] frameworks) and oxygen interstitials (*O*_in_) act as efficient charge capturing centers. These defects, characterized by their thermodynamic trap levels (*E*_T_) and relaxation energies (*E*_R_), were directly linked to critical reliability issues observed in prototype Bi_2_O_2_Se/Bi_2_SeO_5_ FETs. We showed that these oxygen-related defects are the physical origins of both BTI and hysteresis. These results highlight the role of intrinsic dielectric defects in the long-term stability and reliability of Bi_2_O_2_Se-based devices.

## Methods

### Computational Setup

We performed DFT calculations using the Gaussian plane wave (GPW) method as implemented in the cp2k code.^49^ A double-ζ Gaussian basis set was employed in combination with the Goedecker–Teter–Hutter (GTH) pseudopotentials.^50,51^ Geometry optimizations were conducted for structures in various charge states using the nonlocal hybrid functional PBE0-TC-LRC, with a mixing factor α = 0.25 for Hartree–Fock (HF) exchange, to accurately describe the electronic wave function and charge localization. This derivative of the PBE0 functional has been successfully employed for investigations of charge trapping in a variety of crystalline and amorphous systems.^32,43,52–54^ In addition, the auxiliary basis set cFIT9 ^55^ was used to make the calculations of the Hartree–Fock exchange, necessary for hybrid functionals, computationally more feasible. The limited-memory Broyden–Fletcher–Goldfarb–Shanno (LBFGS) algorithm was used to perform geometry optimizations in different charge states. At each geometry-optimization step, the total energy was converged self-consistently using EPS_SCF = 1.0 × 10^–6^ Ha in both the inner and outer SCF loops, corresponding to 2.72 × 10^–5^ eV. Structural relaxation was considered converged when a maximum force of 0.0231 eV/Å, an RMS force of 0.0154 eV/Å, a maximum displacement of 1.59 × 10^–3^ Å, and an RMS displacement of 7.94 × 10^–4^ Å were satisfied. Furthermore, spin polarization was fully included in all DFT calculations through the unrestricted Kohn–Sham formalism to accurately capture the exchange splitting and ground-state energies of defects with unpaired electrons. Additionally, an energy cutoff of 800 Ry together with a relative cutoff of 60 Ry was used for the GPW representation of the electron density.

We derive thermodynamic transition levels from the formation energies for each defect in all possible charge states and determine the most stable charge state of a defect depending on the Fermi level. The formation energy of a vacancy V_*X*_ (*X* = Bi, Se, O) in charge state *q* is given by

1 $$E^{f}\left(V_{X}^{q}\right)=E_{tot}\left(V_{X}^{q}\right)-E_{tot}^{bulk}+\mu_{X}+qE_{F}+\Delta^{q}$$

where *E*_tot_(*V*_X_^*q*^) is the total energy of the supercell containing a vacancy of the species *X* in charge state *q* and *E*_tot_^bulk^ is the total energy of the defect-free structure in the same supercell. The term μ_X_ is the chemical potential needed to add or remove atoms of kind *X* to the bulk to create the defect. μ_O_ is referenced to the total energy per atom of an isolated O_2_ molecule $\left(\mu_{O}^{0}=\frac{1}{2}E_{tot}\left(O_{2}\right)\right)$ , and μ_Se_ is referenced to the total energy per atom of Se (μ_Se_^0^ = *E*_tot_(Se)). The Fermi level (*E*_F_) is calculated relative to the VBM of the oxide *E*_F_ = *E*_VBM_ + ϵ_F_, where *E*_VBM_ can be approximated by the highest occupied Kohn–Sham orbital of the defect-free bulk system.^56^ The correction term Δ^*q*^ was calculated using the FNV-image charge-correction scheme as implemented in the sxdefectalign code, which accounts for the anisotropic dielectric permittivity tensor and the nonbulk nature of our system.^57–59^

### Experimental Setup

Hysteresis and BTI measurements were performed in fixed current-range mode under controlled sampling conditions to ensure a well-defined temporal resolution. In addition, transfer characteristic (*I*_D_(*V*_G_)) measurements were recorded in autorange mode and used for electrostatic modeling. All measurements were conducted using a Keithley 2636B source-measure unit (SMU) system with tungsten probe tips. The devices were measured in a Lakeshore vacuum probe station operated at pressures below 2 × 10^–6^ Torr and in complete darkness to suppress photoinduced effects. Prior to electrical characterization, the samples were stored at room temperature in darkness under vacuum conditions below 1 mbar to minimize environmental degradation. The Lakeshore vacuum probe station was pumped for more than 12 h prior to measurement to ensure a stable high-vacuum environment. For temperature-dependent measurements, each temperature step was allowed to stabilize for approximately 1 h before data acquisition. In addition, hysteresis and PBTI measurements were preceded by electrical preconditioning cycles to reduce the influence of device history and stabilize the initial trap occupancy.

### Transistor Trap-Band Extraction

The extraction of the transistor trap band was performed using the ESiD framework in combination with drift-diffusion-based transient device simulations carried out with the Minimos-NT TCAD simulator.^60,61^ NMP traps were uniformly distributed throughout the gate oxide and across the relevant energy ranges in *E*_T_ and *E*_R_. Device simulations were used to determine the hysteresis response associated with individual traps under the experimentally applied readout current and stress conditions.

Within the charge-sheet approximation, the threshold voltage shift associated with an individual trap depends solely on its charge state and its position within the oxide and is expressed as

2 $$\delta V_{th}=\frac{qt_{OX}}{\epsilon_{0}\epsilon_{ox}A}\left(1-\frac{X}{t_{OX}}\right)$$

where *q* denotes the trap charge, *X* is its distance from the channel interface, *t*_OX_ is the oxide thickness, ϵ_ox_ is the relative permittivity of the oxide, and *A* is the effective sheet area. Under the assumption of defect concentrations not being excessively large, interactions between traps are neglected. Because these discrete point defects are present at sufficiently low concentrations, they do not globally degrade or significantly modulate the macroscopic bulk dielectric constant (κ ≈ 22) of the oxide layer. This allows the total threshold voltage shift to be approximated by the linear superposition of individual trap contributions.

The measured voltage drift is decomposed by solving an optimization problem subject to non-negativity constraints, yielding weighting factors that correspond to the trap density in the defect parameter space. This procedure results in a trap-band distribution that reproduces the experimentally observed voltage shifts under applied stress conditions.

## Supplementary Material



## Notes

Projected density of states for selenium and oxygen vacancies in Bi_2_O_2_Se; projected density of states for selenium-vacancy-related defects in β-Bi_2_SeO_5_; and material parameters used for TCAD simulations (PDF)
