# Supplementary material for: Reliability and Stability Issues in Bi2O2Se/β-Bi2SeO5 Field-Effect Transistors
Source: ACS Nano. 2026 Jul 14;20(29):20498–512. doi: 10.1021/acsnano.6c00419 (PMC13422258; doi:10.1021/acsnano.6c00419)
Supplement: Supplementary file 1 [file nn6c00419_si_001.pdf]

Supporting Information for:

Reliability and Stability Issues in Bi_2O_2Se/ β-Bi_2SeO_5 Field-Effect Transistors

Mina Bahrami,† Mohammad Rasool Davoudi,† Axel Verdianu,† Pedram Khakbaz,† Dominic Waldhoer,† Christoph Wilhelmer,† Junchuan Tang,‡ Congwei Tan,‡ Aftab Nazir,¶ Yu Zheng,¶ Changze Liu,¶ Hailin Peng,‡ Theresia Knobloch,† Michael Waltl,† and Tibor Grasser*,†

†*Institute for Microelectronics, Technische Universität Wien, 1040 Vienna, Austria*

‡*College of Chemistry and Molecular Engineering, Peking University, Beijing 100871, China*

¶*Huawei Technologies Research and Development Belgium N.V., 3001 Leuven, Belgium*

E-mail: bahrami@iue.tuwien.ac.at;grasser@iue.tuwien.ac.at

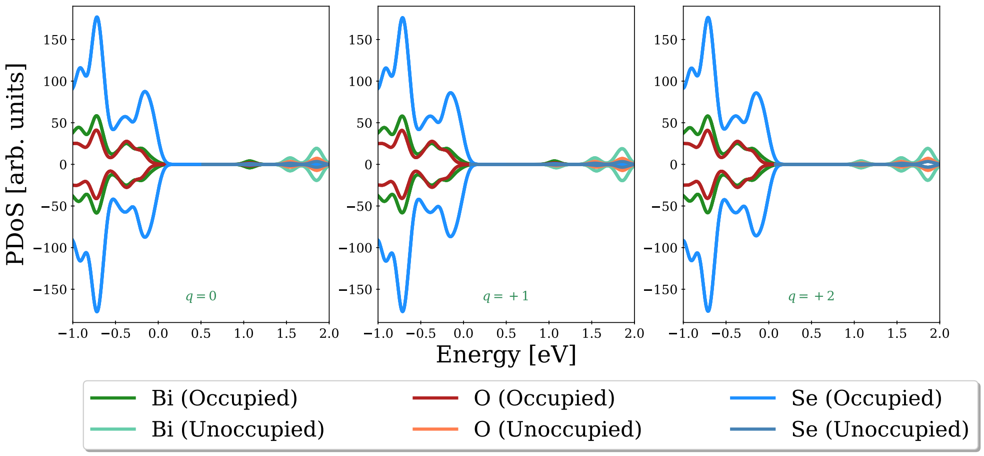

(a)

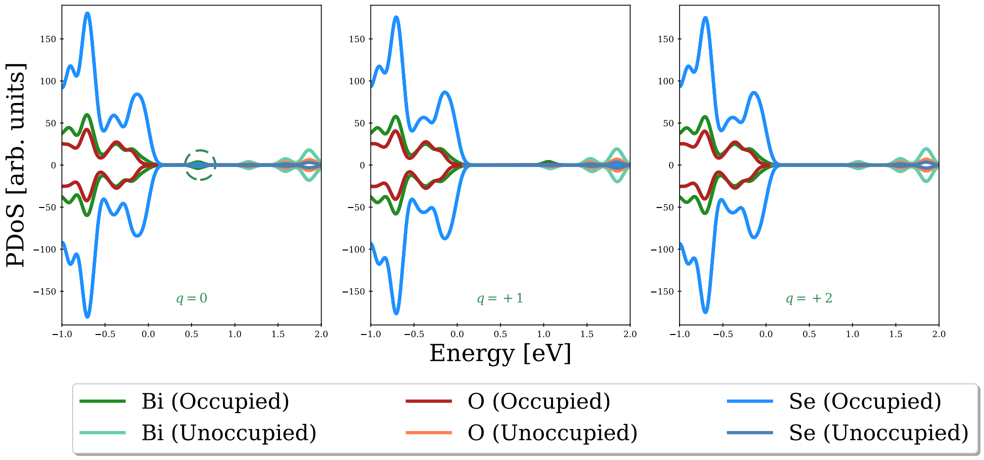

(b)

Figure S1: **(a)** PDoS of a selenium vacancy in Bi_2O_2Se in different charge states. **(b)** PDoS of an oxygen vacancy in Bi_2O_2Se in different charge states.

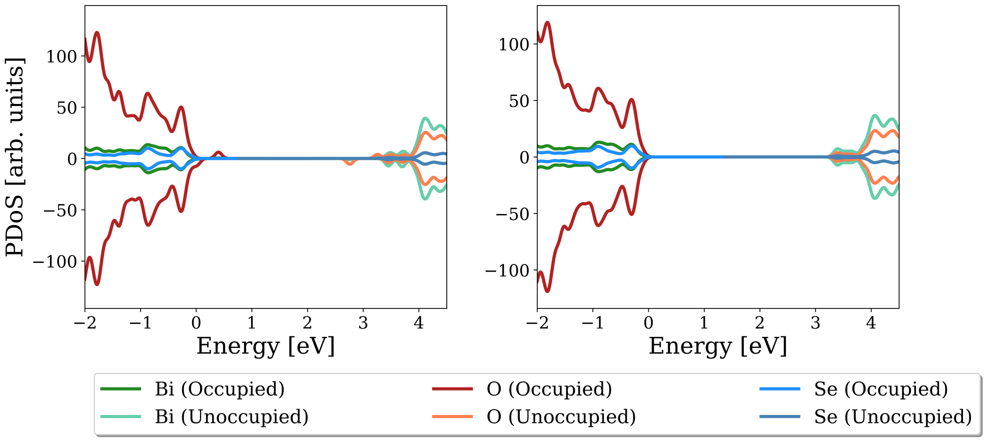

Figure S2: (left) PDoS of a selenium vacancy in Bi_2SeO_5 in the neutral charge state. (right) PDoS of a V_{SeO_2} defect in Bi_2SeO_5 in the neutral charge state.

Material parameters

Table S1: Material parameters of insulator and channel materials that can be determined using both density functional theory (DFT) and experiments, which were used for TCAD simulations

**Segment**	**Parameter**	**Value**
**Insulator – Bi_2SeO_5**	Band Gap (DFT) (E_g)	3.5 eV
	Dielectric Constant	22
	Affinity	2.7 eV
**Channel – Bi_2O_2Se**	Band Gap (DFT) (E_g)	1.09 eV
	Dielectric Constant (ε^c)	99.5[1]
	Mobility	$140\,cm^2/(V\,s)$
	Affinity	4.3 eV
	Effective mass (m_e) (DFT)	0.15 m_0
	Effective mass (m_h) (DFT)	0.15 m_0

References

(1) Khakbaz, P.; Waldhoer, D.; Bahrami, M.; Knobloch, T.; Pourfath, M.; Davoudi, M. R.; Zhang, Y.; Gao, X.; Peng, H.; Waltl, M.; Grasser, T. Two-Dimensional Bi_2SeO_2 and Its Native Insulators for Next-Generation Nanoelectronics. *ACS Nano* **2025**, *19*, 9788–9800.
